# Prostate-specific membrane antigen-radioguided surgery salvage lymph node dissection: experience with fifty oligorecurrent prostate cancer patients

**DOI:** 10.1007/s00345-024-05189-6

**Published:** 2024-08-12

**Authors:** Roman Mayr, Simon Udo Engelmann, Yushan Yang, Maximilian Haas, Thomas Schmid, Marco Julius Schnabel, Johannes Breyer, Daniel Schmidt, Matthias Eiber, Stefan Denzinger, Maximilian Burger, Dirk Hellwig, Jutta Moosbauer, Jirka Grosse

**Affiliations:** 1 Department of Urology, St. Josef Medical Center, University of Regensburg, Landshuter Straße 65, 93053 Regensburg, Germany; 2 Department of Nuclear Medicine, University of Regensburg, 93053 Regensburg, Germany; 3 Department of Nuclear Medicine, School of Medicine, Technical University Munich, Munich, Germany

**Keywords:** Prostate cancer, Oligometastasis, Metastasis directed therapy

## Abstract

**Purpose:**

The higher detection efficacy of PSMA PET for oligometastatic recurrence of prostate cancer has promoted new loco-regional treatment options. PSMA-targeted radioguided surgery (PSMA-RGS) was introduced to facilitate salvage surgery of small tumor deposits. The objectives of this retrospective analysis are to describe an independent single-center consecutive cohort of patients undergoing PSMA-RGS and to evaluate its clinical and oncological outcomes.

**Method:**

Between 2018 and 2022, 53 patients were treated with PSMA-RGS and 50 patients were available for final analyses. All patients were initially treated with radical prostatectomy (RP) and presented with biochemical recurrence (BCR) with at least one positive lesion on PSMA-PET imaging. After preparation of 99mTc-PSMA-I&S and intravenous injection, surgery was performed by using a gamma-probe intraoperatively.

**Results:**

Median age was 70 years (IQR 65–73) and the median PSA at salvage surgery was 1.2 ng/mL (IQR 0.6-3.0). In all patients pathologically positive lesions could be removed during PSMA-RGS. 29 (58%) patients had one pathologically positive lesion, 14 (28%) had two and 7 (14%) had three or more, respectively. The overall complication rate was 26% with 4 (8%), 1 (2%), and 8 (16%) having Clavien-Dindo (CD) type I, II, and IIIb complications, respectively. During the follow-up period 31 (62%) patients experienced BCR and 29 (58%) received further therapy.

**Conclusions:**

PSMA-RGS is a promising treatment option to enhance salvage surgery in early biochemical recurrence. However, only 42% of the patients treated with PSMA RGS remain without a biochemical recurrence. Further research is mandatory to identify patients, who profit from PSMA-RGS.

**Supplementary Information:**

The online version contains supplementary material available at 10.1007/s00345-024-05189-6.

## Introduction

Treatment of prostate cancer patients throughout all stages is rapidly evolving. For biochemical recurrence after primary treatment prostate-specific membrane antigen positron emission tomography (PSMA PET) has substantially improved lesion detection [1, 2]. It is superior compared to conventional imaging techniques such as computed tomography (CT) and magnetic resonance imaging (MRI), and allows the detection of metastatic soft tissue prostate cancer lesions of only a few millimeters in size [3]. With higher detection rates of oligometastatic recurrence the question of treatment beneficence was raised. In a randomized prospective phase III trial metastasis-directed therapy (MDT) significantly prolonged Androgen deprivation therapy (ADT) free survival [4].

In this evolving field, several treatment options were introduced for patients with oligorecurrent prostate cancer, namely, targeted local treatment techniques such as targeted salvage radiotherapy (SRT) or salvage lymph node dissection (SLND) [5]. ADT and other systemic therapies belong to standard of care (SOC) in this subgroup of patients [6]. The SOC with ADT at its front line has several adverse effects such as decreased bone density, decreased muscle mass, decreased libido and weight gain to only name a few [7]. Therefore a main goal of MDTs is prolonging therapy free survival (TFS) and in order to do so, prolonging biochemical recurrence free survival (BFS) [4].

In 2015 prostate-specific membrane antigen-radioguided surgery (PSMA-RGS) was introduced as a new surgical treatment option for oligorecurrent metastatic prostate cancer to facilitate removal of small tumor deposits [8]. This technique has been evaluated for its feasibility and oncological outcome reporting high rate of PSA-response and substantial treatment free periods in a subset of patients [9–14]. Identification of proper candidates remains a challenge. It has been shown that single lesions and low PSA are the best prognostic predictors [12]. In contrast, the use of routine serum markers have a limited prognostic role [15]. Most publications on PSMA-RGS, however, are published by the same research group, with overlap of patient cohorts [16].

The aims of our study are (1) to present and describe a single-center consecutive cohort of patients receiving PSMA-RGS due to oligorecurrent prostate cancer and (2) to evaluate the clinical and oncological outcomes of this treatment regarding BFS, TFS and complications.

## Patients and methods

### Patients

Between September 2018 and September 2022, 53 patients were treated with PSMA-RGS in St. Josef Medical Center, Regensburg. Of these patients, 3 were excluded (one with primary radiotherapy, one with no FU data available and one patient who received two PSMA-RGS), leaving a study cohort of 50 patients for further analyses. All patients included were initially treated with a radical prostatectomy (RP). In alignment with the EAU guidelines for prostate cancer a PSA-recurrence > 0.2 ng/ml was the indication for PSMA/PET CT imaging [17]. All patients presented with biochemical recurrence (BCR) with one or more positive lesions (lymph node or soft tissue) on PSMA PET/CT imaging. After detection of oligometastatic disease (in our cohort ≤4 lesions) in PSMA-PET/CT imaging, patients were discussed in the interdisciplinary tumor board. In correspondence with the therapy recommendation of the interdisciplinary tumor board, patients were offered stereotactic radiotherapy (when possible) or PSMA-RGS. All patients were free of concomitant treatment at the time of PSMA-RGS.

### Procedure

#### Imaging

Patients with localized recurrence of prostate carcinoma as detected by [^68^Ga]Ga-PSMA-11 PET/CT or [^18^F]F-AlF-PSMA-11 PET/CT were suitable for PSMA-RGS according to the recommendation of an interdisciplinary uro-oncological tumor board. PSMA avid lesions were defined by the visual PSMA uptake of morphologically detectable lymph nodes by two board certified readers with many years of experience in PET/CT diagnostics. An evaluation based on a threshold value of the SUVmax was not performed in the present study, not least because two different radiopharmaceuticals were used.

The day prior to surgery [^99m^Tc]Tc-PSMA-I&S ([^99m^Tc]Tc-mas3-y-nal-k(Sub-KuE) was prepared and quality-checked according to the German Medicinal Products Act (AMG, § 13.2b and § 55) after notification to the legal authority (Government of Oberfranken and Oberbayern, Germany) [18]. To comply with radiation protection regulations, the applied activities were administered as described by Schmidt et al. [19]. Three to four hours after intravenous injection whole-body scintigraphic scans were acquired (LEHR collimation), followed by single photon emission computed tomography/low-dose CT (SPECT/CT) of abdomen and pelvis using a Siemens Intevo Bold SPECT/CT system (Siemens Healthineers, Erlangen, Germany, LEHR collimation). Low-dose CT was used for anatomic localization and attenuation correction. Image analysis and cross-validation with the PSMA PET/CT of the findings were performed using the software syngo.via version VB40 (Siemens Healthineers, Erlangen, Germany) by a board-certified nuclear medicine physician. If the lesion(s) detected by PSMA PET/CT could not be identified on the scans after four hours, SPECT/CT was repeated approximately 22 to 23 h after tracer injection on the day of surgery.

#### Surgery

PSMA-RGS was performed the day after the tracer injection by using the Gamma-Probe (Neoprobe GDS Gamma Detection System, Devicor Medical Products, USA). Before sterile draping of the Gamma-Probe, quality assurance checks in accordance with legal requirements were carried out. The different sites of the PSMA-RGS are depicted in Table 1. PSMA-RGS was performed as open surgery by two experienced urological surgeons (RM and SD). When RGS was implemented in 2018, RM and SD had three years and 15 years of open uro-oncological surgery experience, respectively. The first 10 RGS cases were carried out by both surgeons together. The choice of incision was made dependent on the location of the target lesion, usually via median lower abdominal laparotomy. Lymph node resection was performed in correlation with PSMA PET/CT and SPECT/CT imaging. Radioguidance was achieved through in vivo measurements using the gamma probe with numerical and acoustic feedback as a response to ^99m^Tc. Main objective of the surgery was removing the target lesion(s) detected in PSMA PET/CT. Depending on the anatomical location, the anatomical adjacent structures including vessels, intestine and urinary tract were identified and mobilized to reach the target lesion(s). During this maneuver other surrounding lymph nodes have been resected. After the resection the surgical field was measured with the gamma probe for additional potential lesions. All removed lesions were measured again ex vivo to confirm the successful resection of the [^99m^Tc]Tc-PSMA-I&S-positive metastatic lesion(s).

**Table 1:** Preoperative and postoperative patient characteristics

Characteristic	*n* = 50
Age, median (IQR)	70 (65-73)
T-Stage at RP, *n* (%)	
pT2	21 (42)
pT3a	14 (28)
pT3b	13 (26)
NA	2 (4)
R-Stage at RP, *n* (%)	
R0	35 (70)
R+	9 (18)
Rx	4 (8)
NA	2 (4)
N-Stage at RP, *n* (%)	
N0	41 (82)
N+	7 (14)
Nx	2 (4)
Lymph node yield at RP, median (IQR)	14 (8-20)
Positive lymph nodes at RP, *n* (%)	
0	41 (82)
1	5 (10)
≥2	2 (4)
Unknown	2 (4)
ISUP gleason grade group at RP, *n* (%)	
I, II or III	30 (60)
IV or IV	17 (34)
NA	3 (6)
RT after RP, *n* (%)	
Prostatic fossa	27 (54)
Prostatic fossa and one/both sided pelvic lymph nodes	6 (12)
Time between RP and RGS (mo), median (IQR)	44 (23-92)
PSA prior to RGS (ng/mL), median (IQR)	1.2 (0.6-3.0)
Surgery time (min), median (IQR)	94 (62-139)
Time interval between last PSA and RGS in days, median (IQR)	38 (6-78)
PSA doubling time (months), median (IQR)	5.5 (3.2-8.8)
PSA velocity (ng/ml/year), median (IQR)	1.9 (0.6-4.0)
PSMA-avid lesions in PET/CT, *n* (%)	
1	25 (50)
2	18 (36)
≥3	7 (14)
Location of PSMA-avid lesions in PET/CT, *n* (%)	
Pelvic unilateral	26 (52)
Retroperitoneal	6 (12)
Pararectal/presacral	7 (14)
Perivesical	3 (6)
Pelvic + pararectal/perivesical	3 (6)
Retroperitoneal + other	4 (8)
Abdominal wall	1 (2)
Highest postoperative complications (Clavien-Dindo), *n* (%)	
None	37 (74)
I	4 (8)
II	1 (2)
IIIa	0
IIIb	8 (16)
Pathologically positive lesions after RGS, *n* (%)	
1	29 (58)
2	14 (28)
≥3	7 (14)
Biochemical recurrence after RGS	31 (62)
Salvage therapy type after RGS	
None	21 (42)
RT only	3 (6)
ADT only	7 (14)
New hormonal agent only	2 (14)
ADT+ other	10 (20)
Other combination	5 (10)
BFS (mo) in pts. experiencing BCR during FU, median (IQR)	6 (3-12)
BFS (mo) in pts. not experiencing BCR during FU, median (IQR)	20 (13-39)
TFS (mo) in pts. receiving therapy during FU, median (IQR)	7 (4-13)
TFS (mo) in pts. not receiving therapy during FU, median (IQR)	19 (13-37)

*RP: Radical prostatectomy; IQR: Interquartile range; ISUP: International Society of Urological Pathology; RT: Radiotherapy; RGS: Radioguided surgery; PSA: Prostate-specific antigen; PSMA: Prostate-specific membrane antigen; ADT: Androgen deprivation therapy; BFS: Biochemical recurrence free survival; BCR: Biochemical recurrence; TFS: Therapy free survival

### Outcomes and follow up

Outcomes of interest were TFS, defined as survival without further treatment, and BFS, defined as prostate specific antigen (PSA) < 0.2ng/mL without additional treatment at follow up. BFS and TFS time was calculated in months from the time of PSMA-RGS to the event (BCR or first administered additional therapy after RGS) or end of follow up. Further outcome variables were PSA change after surgery, additional therapy type administered to the patients after RGS, and complications classified according to Clavien-Dindo [20]. Follow-up (FU) data were collected using in-hospital patient records and telephone interviews with patients and local urologists.

### Statistical analyses

All data analyses were performed using SPSS software (Version 29.0; IBM Corp., Armonk, NY, USA). Graphs were created using GraphPad Prism software (version 10.0.2, GraphPad, Boston, MA, USA). Frequencies are presented as absolute numbers and percentages. Continuous data are presented as median with interquartile range (IQR). BFS data was analyzed using univariable Cox regression. Kaplan-Meier curves were used to illustrate BFS and TFS.

### Ethical approval

The retrospective analysis complies with the ethical standards described in the latest declaration of Helsinki; it was approved by the institutional ethics review board of the University of Regensburg (Approval number: 22-2819-101).

## Results

### Preoperative characteristics

In this study 50 patients were included who received PSMA-RGS at a median age of 70 (IQR 65–73) at our center between September 2018 and September 2022. All patients received a radical prostatectomy (RP) as primary treatment with a median of 44 months (IQR 23–92) before PSMA-RGS (Table 1). At primary RP 48 (96%) of patients received simultaneous primary bilateral lymphadenectomy (LND). Of these, 35 (70%) received extended LND, 10 (20%) received limited LND and in 3 (6%) patients LND template is unknown at primary RP, respectively. The median PSA prior to PSMA-RGS was 1.2 ng/mL (IQR 0.6-3.0). At RP 7 (14%) and 10 (20%) patients were diagnosed with a Gleason grade group of IV and V respectively and 14 (28%) and 13 (26%) had a T3a and T3b stage prostate cancer in pathology results at RP, respectively. Positive lymph nodes were found in 7 (14%) patients, 9 (18%) had positive surgical margins and 33 (66%) received adjuvant radiotherapy after RP. In PSMA PET/CT imaging prior to PSMA-RGS 25 (50%) had one PSMA-avid lesion and 25 (50%) had two or more lesions. The location distribution of PSMA-avid lesions is depicted in Table 1.

### Postoperative outcomes

**Fig. 1:**
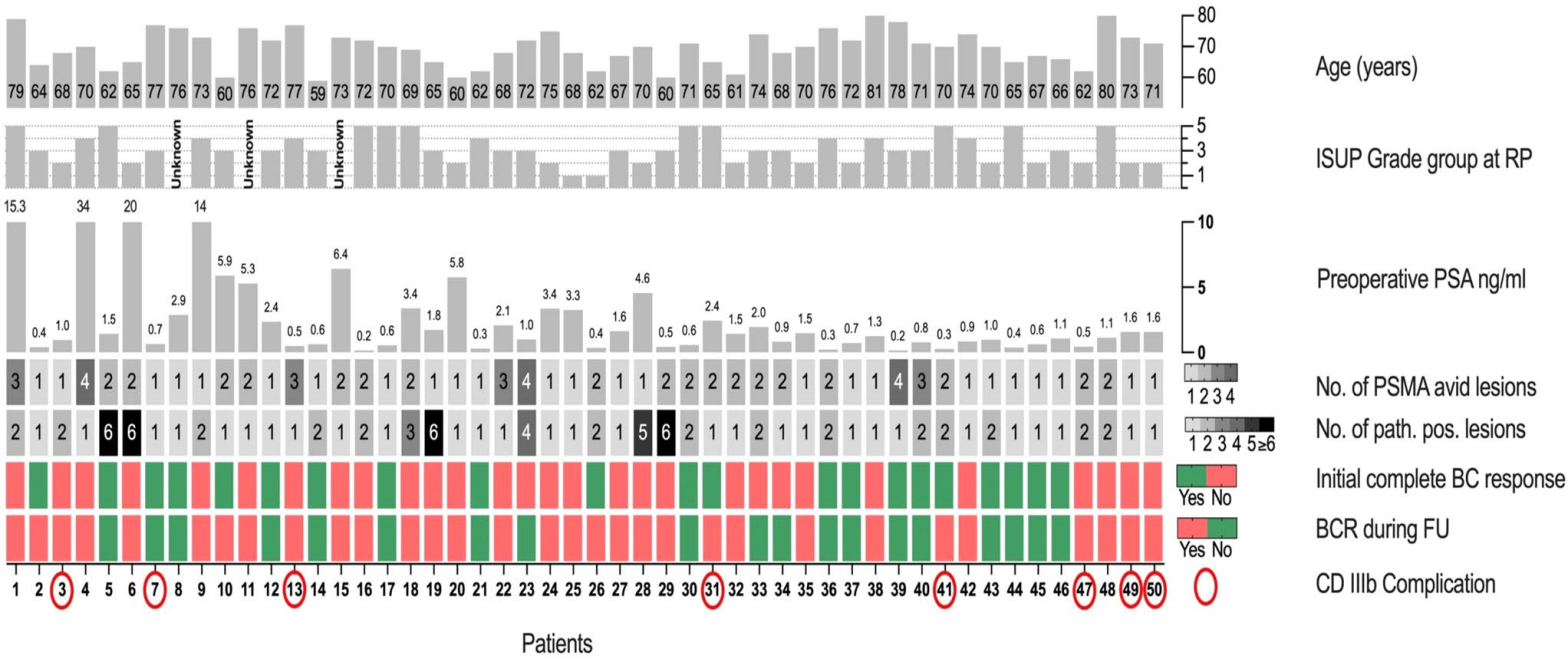
Depiction of different pre- and postoperative variables of individual patients (X-axis) included in our study. ISUP: International Society of Urological Pathology; RP: Radical prostatectomy; No.: Number; PSMA: Prostate-specific membrane antigen; path. pos.: pathologically positive; BC: Biochemical; BCR: Biochemical recurrence; FU: Follow-up; CD: Clavien-Dindo

In all patients pathologically PSMA-positive lesions could be removed by PSMA-RGS, of these 29 (58%) had one pathologically positive lesion, 14 (28%) had two and 7 (14%) had three or more positive lesions (Table 1). The count of lesions removed ranged from 1 to 21 lesions, of which positive lesions ranged from 1 to 13. Of all lesions removed during PSMA-RGS a median of 52.3% (IQR 33.3-100%) were positive. Five (10%) of patients had minor complications (Clavien-Dindo [CD] I and II) and 8 (16%) had CD III complications. No type IV complication or higher occurred in the entire cohort. Figure 1A summarizes pre- and postoperative variables of the individual patients.

**Fig. 2:**
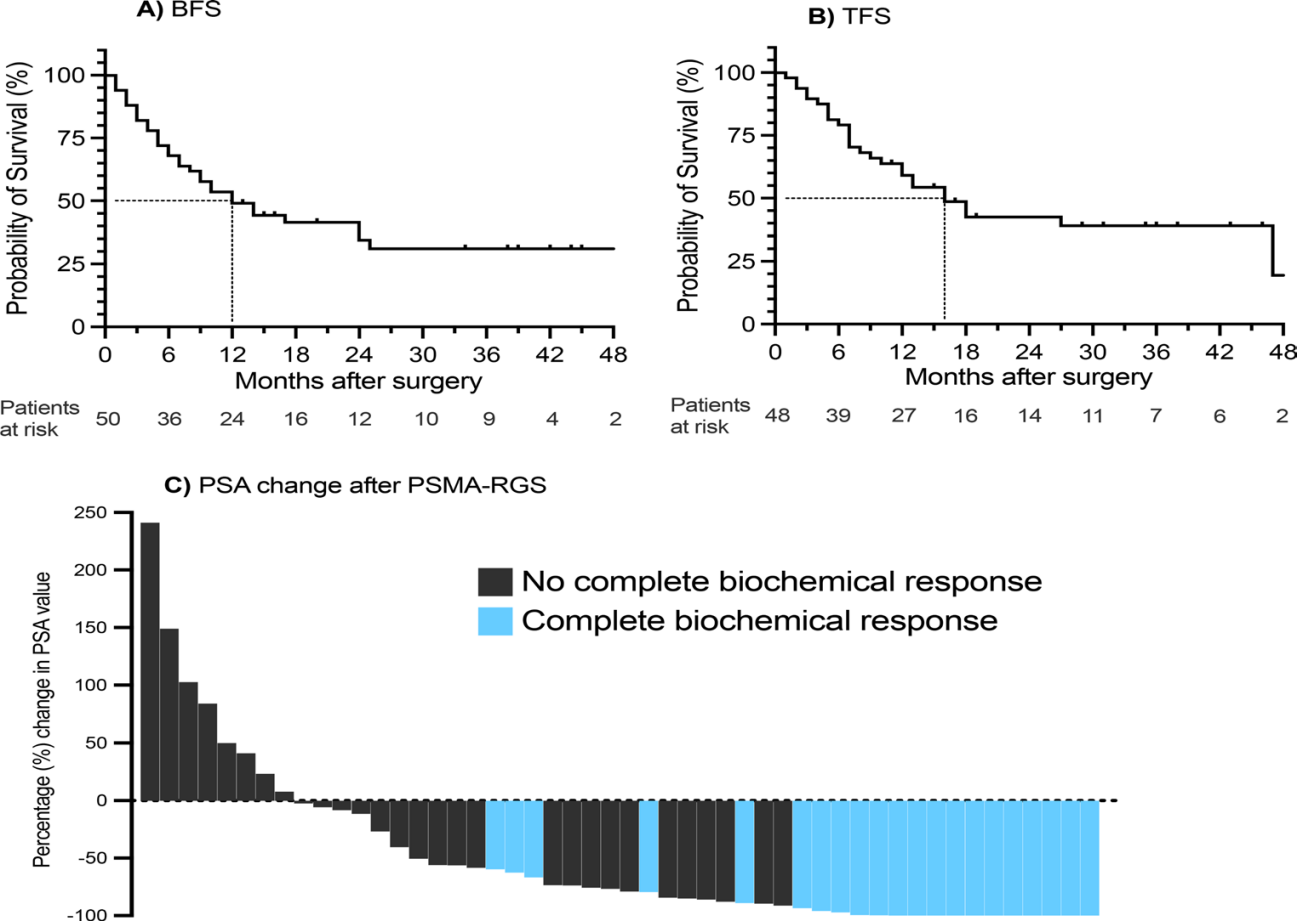
**A**) and **B**) Kaplan-Meier curves showing biochemical recurrence-free survival (BFS, ‘**A**’) and therapy-free survival (TFS, ‘**B**’). **C**) Waterfall plot showing the percentage change of PSA value after prostate-specific membrane antigen-radioguided surgery (PSMA-RGS) for all 50 patients, and indicating those with initial complete biochemical response

Initial complete response (defined as PSA < 0.2ng/ml in the first measurement after PSMA-RGS) was recorded in 21 (42%) patients. In the entire cohort, the median TFS was 13 months (IQR 6–21), and the median BFS was 10 months (IQR 5–21), respectively. However, 21 (42%) patients were free of therapy, and 19 (38%) did not experience BCR at last follow-up. Of those who experienced BCR, the median time to BCR was 6 months (IQR 3–12). Two patients died during the follow-up period, both after experiencing both events (BCR and salvage therapy). Figure 2A&B shows Kaplan-Meier curves for BFS and TFS, depicting a median of 12 months (95% confidence interval [CI]: 6.8–17.2) for BFS and 16 months (95% confidence interval [CI]: 10.5–21.5) for TFS for the entire cohort. The percentage change in PSA value after PSMA-RGS is depicted in Fig. 2C.

In univariable Cox regression analyses for BFS, the preoperative PSA value (continuous) was found to be a significant predictor of BCR (HR: 1.1; 95% CI: 1.04–1.16; *p* < 0.01), (Table 2). Apart from this, no significant predictors could be found in univariable cox regression analysis (see Table 2).

**Table 2:** Univariable cox regression analysis for biochemical recurrence after PSMA-RGS (events *n* = 31)

	Univariable analysis		
Characteristic	HR	95% CI	*P*-Value
Age (continuous)	1.00	0.94–1.06	0.86
T-Stage at RP (Ref. pT2)			
pT3a	0.75	0.23–1.74	0.50
pT3b	0.50	0.19–1.32	0.16
R-Stage at RP (Ref. R0)			
R+	0.56	0.19–1.62	0.28
Rx	0.24	0.03–1.76	0.16
N-Stage at RP (Ref. N0)			
N+	0.15	0.02–1.08	0.06
Lymph node yield at RP (Ref. 0)			
1–10	0.85	0.26–2.84	0.80
11–20	0.75	0.24–2.37	0.63
≥ 21	0.67	0.19–2.38	0.53
ISUP gleason grade group at RP (ref. I-III)			
IV or V	0.93	0.44–1.96	0.84
Time between RP and RGS (mo) (continuous)	1.00	0.10–1.01	0.29
PSA prior to RGS (ng/ml) (continuous)	**1.10**	**1.04–1.16**	**< 0.01**
PSA doubling time (months) (continuous)	0.96	0.90–1.02	0.252
PSA velocity (ng/ml/year)	**1.02**	**1.01–1.04**	**0.006**
PSMA PET-avid lesions (Ref. 1)			
2	1.17	0.55–2.50	0.69
3	1.54	0.43–5.44	0.51
4	0.54	0.07–4.13	0.56
PSMA PET-avid lesion (Ref. 1)			
2–4	1.37	0.56–2.32	0.72
Location of PSMA PET-avid lesions (ref. Pelvic)			
Retroperitoneal	1.51	0.50–4.53	0.46
Retroperitoneal + pelvic or other	0.90	0.41–1.97	0.78
Pathologically positive lesions after RGS (ref. 1–2)			
≥ 3	1.05	0.40–2.73	0.93

## Discussion

PSMA-RGS has emerged as an interesting application for locoregional surgical treatment of oligometastatic disease. However, data on efficacy, feasibility and complications are still limited with most publications deriving from one single group [8, 16]. Our study analyzed data of 50 consecutive patients treated with PSMA-RGS due to BCR of prostate cancer at a single tertiary academic center.

In the past, SLND was critically discussed in literature in respect of long-term oncological outcomes [21, 22]. However, these discussions were concerning standard SLND without the use of RGS. Literature on PSMA-RGS has shown that patients with PSMA-avid lesions at BCR benefit from this surgical treatment in several ways. Knipper et al. showed that they were able to remove 95% of PSMA-avid lesions, opposed to studies on standard SLND where pathology revealed no metastatic tissue in 20% [9, 21]. Our findings confirm and support the findings of high removal rates of metastatic tissue. In our study in all patients (100%) one or more pathologically positive lesion could be removed during PSMA-RGS.

The largest cohort study on PSMA-RGS by Knipper et al. states a 60%-rate of complete early biochemical response [9]. They define this as PSA < 0.2 ng/mL without further treatment at 2–16 weeks after PSMA-RGS. Using this definition in our study, 42% of patients have a complete early biochemical response. However, we find this term and definition misleading. It does not give any information on medium or long-term effect. The endpoint, which in our view is much more important, is BFS. Here Knipper et al. report a third of patients not having BCR at 2-year follow-up. Similarly in our study 38% of patients had no BCR at a median follow-up of 20 months. As depicted in Fig. 1, which demonstrates individual patient characteristics, complete early biochemical response does not necessarily mean that the patient will not experience BCR and vice versa.

For obvious reasons TFS correlates strongly with BFS. In our study 42% of patients had no salvage therapy after PSMA-RGS at a median FU of 19 months. TFS translates into better quality of life due to missing side effects of further therapies, especially ADT, with which most patients with BCR are treated solely or in a combination with other therapies. Adverse effects of these therapies, such as fatigue, cardiovascular disease or osteoporosis are well known and can be severe [23].

Adverse effects of PMSA-RGS must also be discussed. Knipper et al. described < 7% higher-grade complications in their study and 32% with complications of any grade [9]. In our study we found a higher rate of grade III CD complications at 16%, but a lower percentage of overall complications with 26%. Nonetheless, none of our patients suffered a grade IV or higher complication and all complications were successfully treated, with most grade III complications being either hydronephrosis treated with temporary ureteral stenting (double-J stent), secondary wound herniation or postoperative wound healing disturbance (Supplementary Table 1). PSMA-RGS is already being carried out as a robotic surgical intervention with a drop-in Gamma probe. The first prospective feasibility study by Barros et al. shows very promising results, especially regarding complication rates with only 1 of 21 patients suffering a grade III complication [24].

Horn et al. have shown that low PSA and single lesions on PSMA-PET/CT are significant predictors for a favorable BFS [12]. In univariable analysis we could demonstrate that low PSA was a significant predictor (HR 1.1; 95% CI 1.04–1.16; *p* < 0.01) of favorable BFS, while single lesions on PSMA PET/CT were not (HR 1.37; 95%CI 0.56–2.3; *p* = 0.72). As demonstrated in Fig. 1 all patients with no biochemical recurrence during follow up had a PSA-Value < 3ng/ml. In contrast, of 19 patients that were free of biochemical recurrence, 8 harbored more than one PSMA-avid lesion and 4 patients had an extrapelvine (paraortal, interaortocaval, abdominal wall) lesion. We therefore conclude from our data, that a low PSA is the only reliable prognostic factor for a favorable course after RGS. However, other studies have shown, that localization and number of PSMA-avid lesions can be predictive factors for a favorable outcome [5, 9]. We have renounced to carry out multivariable analysis due to our limited number of 31 events. Apart from clinical predictors such as PSA value, localization and number of PSMA-avid lesions there have been indications for molecular and genetic predictors of clinical outcome in metastatic prostate cancer [25, 26]. Further investigations should be made to identify possible genetic and molecular outcome predictors in the context of PSMA-RGS.

Our study has several limitations. Although being one of the largest single-center studies on this treatment published to this date, our study is still limited by cohort size. Patients with oligometastatic recurrence in PSMA-PET/CT imaging were included in this study. Due to the nature of PSMA-PET/CT imaging there is a certain detection bias, affecting the results and outcome after PSMA-RGS. Examples of detection limitation of PSMA-PET/CT imaging can be seen in patients 5, 6, 19, 28 and 29 (Fig. 1), with only one or two PSMA avid lesions in imaging but all with five or more positive lesions in the pathological assessment. In addition, there was no central pathology review. Due to the retrospective nature of our study, there was no standardized FU and therapy protocol after PSMA-RGS, which may have affected the results. Only a limited number of patients, who experienced biochemical recurrence, had a PSMA-PET/CT, which should be addressed in future trials. Patient treatment was left to the discretion and experience of outpatient urologists. Also, our study is lacking a control group or comparison groups treated with other MDTs such as salvage stereotactic RT. PSMA guided salvage RT for nodal recurrence exists and has been associated with similar results compared to PSMA-RGS regarding BFS [27]. As already suggested by Knipper et al. a randomized controlled trial investigating observation vs. systemic treatment vs. RT vs. surgery would be desirable [9]. Although the surgeons who performed PSMA-RGS were experienced open uro-oncological surgeons, a learning curve considering PSMA-RGS itself may affect the results.

## Conclusions

PSMA-RGS is a promising new procedure to facilitate surgical removal of small tumor deposits especially in early BCR. However, as a substantial portion of patient experience recurrence within a limited time frame, careful patient selection through close interdisciplinary collaboration between urologists and nuclear medicine physicians and detailed informed consent is crucial for this treatment. Our study confirms and supports the findings made by the limited number of studies published so far and should encourage the initiation of larger prospective studies.

## Electronic supplementary material

Below is the link to the electronic supplementary material.

Supplementary Material 1

## Data Availability

All data analyzed during the current study are available from the corresponding author on reasonable request.
