# Supplementary material for: Prostate-specific membrane antigen-radioguided surgery salvage lymph node dissection: experience with fifty oligorecurrent prostate cancer patients
Source: World J Urol. 2024 Aug 12;42(1):483. doi: 10.1007/s00345-024-05189-6 (PMC11319506; doi:10.1007/s00345-024-05189-6)
Supplement: Supplementary file 1 — Supplementary Material 1 [file 345_2024_5189_MOESM1_ESM.docx]

Supplementary Information

**Supplementary Table 1.** Detailed information of Clavien-Dindo IIIb complications in our study cohort.

| **Patient** | **Location target lesion** | **Complication** |
| --- | --- | --- |
| 3 | left dorsal of bladder | Injury of left ureter with consecutive hydronephrosis requiring ureteral stenting five days after PSMA-RGS. Three months later the ureteral stent was removed without further therapy needed. |
| 7 | Right iliac vessels | Fascial dehiscence 3 days after PSMA-RGS requiring revision surgery with fascial suture. |
| 13 | Right iliac vessels | The patient presented with right hydronephrosis 5 months after PSMA-RGS. Computed tomography suspected a malignancy in the distal ureter. Ureterorenoscopy diagnosed a stricture due to scarring. No malignancy was detected. Temporary treatment was achieved by nephrostomy. Permanent therapy was then achieved by ureter re-implantation surgery. |
| 31 | Right iliac vessels | Fascial dehiscence 2 days after PSMA-RGS requiring revision surgery with fascial suture. |
| 41 | presacral | During PSMA-RGS the left ureter had to be re-implanted due to iatrogenic injury. |
| 47 | Left iliac vessels | The patient presented 8 days postoperatively for readmission due to a subcutaneous hematoma. Surgical removal of the hematoma, and renewed wound suture was performed. |
| 49 | Right iliac vessels | The patient presented with right hydronephrosis 2 months after PSMA-RGS, requiring ureteral stenting. |
| 50 | Right iliac vessels | Readmission due to fascial dehiscence 11 days postoperatively. Fascial- and wound suture was performed. |
